# The research landscape of the quality of life or psychological impact on gynecological cancer patients: A bibliometric analysis

**DOI:** 10.3389/fonc.2023.1115852

**Published:** 2023-02-07

**Authors:** Jiayuan Zhao, Yujia Kong, Yang Xiang, Junjun Yang

**Affiliations:** National Clinical Research Center for Obstetric & Gynecologic Diseases/Department of Obstetrics and Gynecology, Peking Union Medical College Hospital, Chinese Academy of Medical Sciences & Peking Union Medical College, Beijing, China

**Keywords:** quality of life, psychologic, genital neoplasms, female, bibliometrics, VOSviewer, CiteSpace

## Abstract

**Background:**

Gynecological cancer is one of the most common cancers in women. The quality of life (QoL) or psychological impact has emerged as an outcome indicator in many clinical trials of gynecological cancer and gained much concern in the clinical setting at the start of the 21st century. Our paper conducted a bibliometric analysis of QoL or psychological impact on gynecological cancer patients to show the status and hotspots.

**Methods:**

Related publications from 2000 to 2022 were included by screening from the Web of Science Core Collection (WOSCC) on 26 June 2022. The bibliometrics was analyzed and visualized by bibliometrix R-package, VOSviewer, and CiteSpace V.

**Results:**

A total of 6,479 publications were included in our study. The publications in this field were increased annually. The United States (n = 2,075) was the country with the most published papers. Sydney University (n = 167) was the most productive affiliation. *Gynecologic Oncology* and *Journal of Clinical Oncology* were the most relevant and most cited sources, respectively. The article written by Bray F et al. has the highest citation. Kim J and Aaronson NK ranked first in most productive author and most co-cited author, respectively. The keywords “mortality”, “fertility preservation”, and “palliative care” have bursts till 2022, which represented the frontiers of this field.

**Conclusion:**

Our study provides an overall analysis of QoL or psychological impact on gynecological cancer patients, which can serve as a reference in future research.

## 1 Introduction

Gynecological cancer mainly consists of cervical cancer, endometrial cancer, ovarian cancer, and gestational trophoblastic neoplasia. In 2020, gynecological cancer accounted for 14.4% of new cancer cases of women among 9.2 million new cases (source: GLOBOCAN 2020). Cervical cancer is the fourth most common cancer in women, followed by endometrial cancer ranking sixth, and ovarian cancer ranking eighth (1). During treatment, gynecological cancer patients often need to undergo surgery, chemotherapy, radiotherapy, targeted immunotherapy, etc. They have to cope with the side effects of these treatments, which undoubtedly cause physical and psychological impacts on them. The postoperative complications include infections such as febrile morbidity, pneumonia, and urinary tract infection and non-infection symptoms such as lymphocyst, ureteral fistula, and premature ovarian failure (2). The chemotherapy and radiotherapy complications can affect the hematological system (i.e., leukopenia, anemia, and thrombocytopenia), digestive system (i.e., indigestion, nausea, vomiting, and diarrhea), genitourinary system (sexual dysfunction, urinary frequency, and incontinence), respiratory system (dyspnea), and skin (i.e., hair loss and skin rash) and cause sleep disorders and so on. The psychological impact includes anxiety and depression or even post-traumatic stress disorder (3–5). In addition to the side effects of the treatment, with the progression of gynecological cancer, patients may suffer from cancer cachexia syndrome, which is characterized by specific energy metabolism alterations and symptoms such as fatigue, anorexia, nausea, anemia, and immunodepression (6). These complications cause heavy burdens on gynecological cancer patients, reduce their quality of life (QoL), or cause psychological problems without remedy (7). With the variety of treatment options and drug combinations available, many clinical trials evaluate QoL and psychological impact as outcome indicators, which help clinical decision making (6, 8–11). As researchers point out, after treatment for locally advanced cervical cancer, worsening of QoL was observed in sexual enjoyment, peripheral neuropathy, and menopausal symptoms. Moreover, the neoadjuvant chemotherapy group experienced a lower burden of menopausal symptoms and higher scores in sexual/vaginal functioning than the chemoradiation group (11).

Many instruments and scales are applied to help gynecologists assess their patients’ QoL or mental health, such as the European Organisation for Research and Treatment of Cancer Quality of Life Questionnaire 30 Version 3 (EORTC QLQ-C30), Hospital Anxiety and Depression Scale (HADS), Female Sexual Function Index (FSFI), and Functional Assessment of Cancer Therapy-General Version 4 (FACT-G). These questionnaires are usually used in combination to score patients (12–14).

Bibliometrics analyzes publication records and presents them visually through the application of mathematical and statistical methods (15, 16). Bibliometric analysis relies on science mapping tools, such as CiteSpace (17–19), VOSviewer (20), and HistCite (21).

Since the start of the 21st century, more and more articles and reviews have emerged on QoL or psychological impact on gynecologic oncology patients. In this paper, we did a bibliometric analysis to identify the global trends, the network of collaboration, research directions, etc., of this field from 2000 to 2022. As we know, this is the first bibliometric analysis targeted at QoL or the psychologic impact of gynecologic oncology patients.

## 2 Methods

### 2.1 Data collection

On 26 June 2022, an advanced document retrieval was performed on the Web of Science Core Collection (WOSCC database). The publication date was limited from 1 January 2000 to 1 June 2022. The search formula was listed as follows:

#1 ((((((((TS=(Quality of Life)) OR TS=(Life Quality)) OR TS=(Health-Related Quality Of Life)) OR TS=(Health Related Quality Of Life)) OR TS=(HRQOL)) OR TS=(psychological impact)) OR TS=(emotional impact)) OR TS=(Psychological Wellbeing)) OR TS=(mental health)

#2((((((TS=(gynecology oncology)) OR TS=(gynecologic cancer)) OR TS=(gynecologic neoplasm)) OR TS=(cervical cancer)) OR TS=(ovarian cancer)) OR TS=(endometrial cancer)) OR TS=(gestational trophoblastic neoplasia)

Publication date 2000-01-01 – 2022-06-01

#1 AND #2

**Figure 1:**
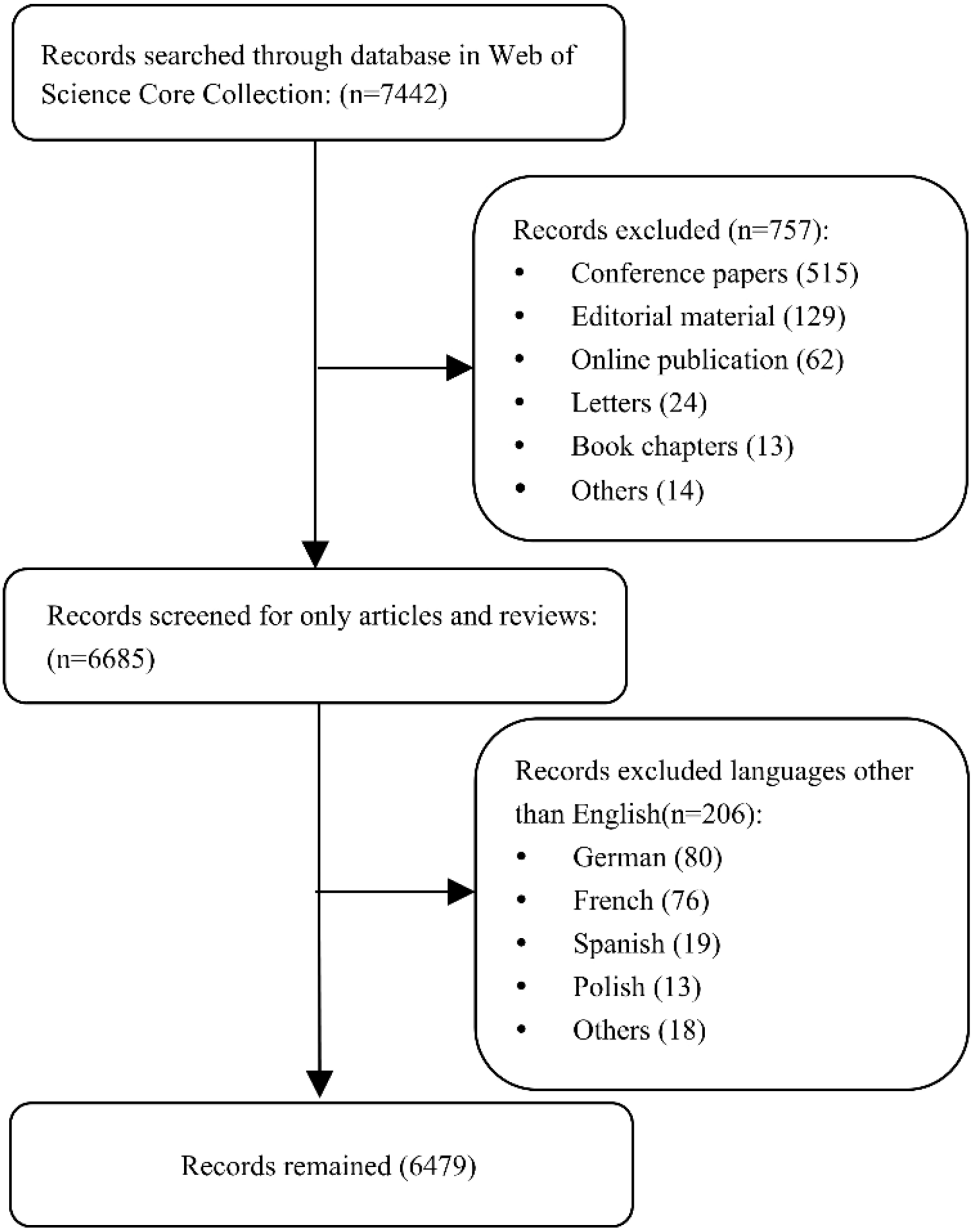
The process of record selection.

The detailed search process is outlined in **Figure 1**. Articles and reviews were included in this bibliography analysis, and the language was restricted to English. Eventually, a total of 6,479 publications were selected and exported in plain text file format for full record and cited reference.

### 2.2 Visualization and statistical tools

Bibliometrix R-package (http://www.bibliometrix.org) provides tools for quantitative bibliometrics and scientometrics research. It runs on RStudio v.4.1.2 software (RStudio, PBC, Boston, MA, USA). It can produce a descriptive analysis of a bibliographic data frame, as well as build a network for analyzing bibliographic coupling, co-citation, collaboration, and co-occurrence (22). We used it to extract the most relevant countries, journals, and locally cited publications as tables. RStudio was also used to conduct the geographic visualization analysis of country collaboration.

VOSviewer (version 1.6.18, the Centre for Science and Technology Studies at Leiden University (Netherlands)) is a software tool for the creation of maps based on network data and for visualizing and exploring these maps. VOSviewer can be used to construct networks of publications, journals, authors, research institutions, countries, keywords, or terms. With VOSviewer, bibliographic database files (such as Web of Science files) can be used to build a network. This software can be freely downloaded from www.vosviewer.com (20). In this analysis, we constructed a geographic map of countries and institutional networks based on the co-authorship links, and a publications network based on the citation links. The world borders of the geographic map were downloaded from https://www.tudelft.nl/en/librarv/research-analvtics/case-21-tu-delft-top-collaborators-2. Based on Clement Lev’s “Map of Countries” plugin for Gephi software, graph nodes are used to represent the country borders and their coordinates (23).

CiteSpace V6.1.R2 (Drexel University, Philadelphia, PA, United States) is a Java application for analyzing and visualizing co-citation networks (24). It can be freely downloaded from http://cluster.cis.drexel.edu/~cchen/citespace/download/. The new version 6.1.R2 was built on 20 June 2022. It can be used to build a cooperative network of authors, institutions, or countries; co-occurrence analysis of terms, keywords, or categories; co-citation analysis of references, authors, or journals; and coupling analysis of the paper (17). In this analysis, we use CiteSpace software to draw the dual-map overlay of journals, citation bursts of the top 20 reference and top 20 keywords, a network of authors, and the timeline view of the keywords. The parameter settings were set in the following: the timespan: 2000–2022 (Slice Length = 2), selection criteria: top 50 per slice, link retaining factor (LRF) = 3.0, look back years (LBY) = 5, e for top N (e) = 1.0.

GraphPad Prism 9.0 was used to draw the line graph of annual production and perform a linear regression based on this line graph. The coefficient of determination (R^2^) represents the proportion of variance in the dependent variable that can be predicted. The closer the R^2^ value is to 1, the better the fitting; and the closer it is to 0, the worse the fitting.

## 3 Results

### 3.1 Distribution of included publications

In total, 6,479 publications were included in our study with the timespan from 2000 to 2022. There were 5,092 articles and 1,387 reviews, which account for 78.59% and 21.41%, respectively.

**Figure 2:**
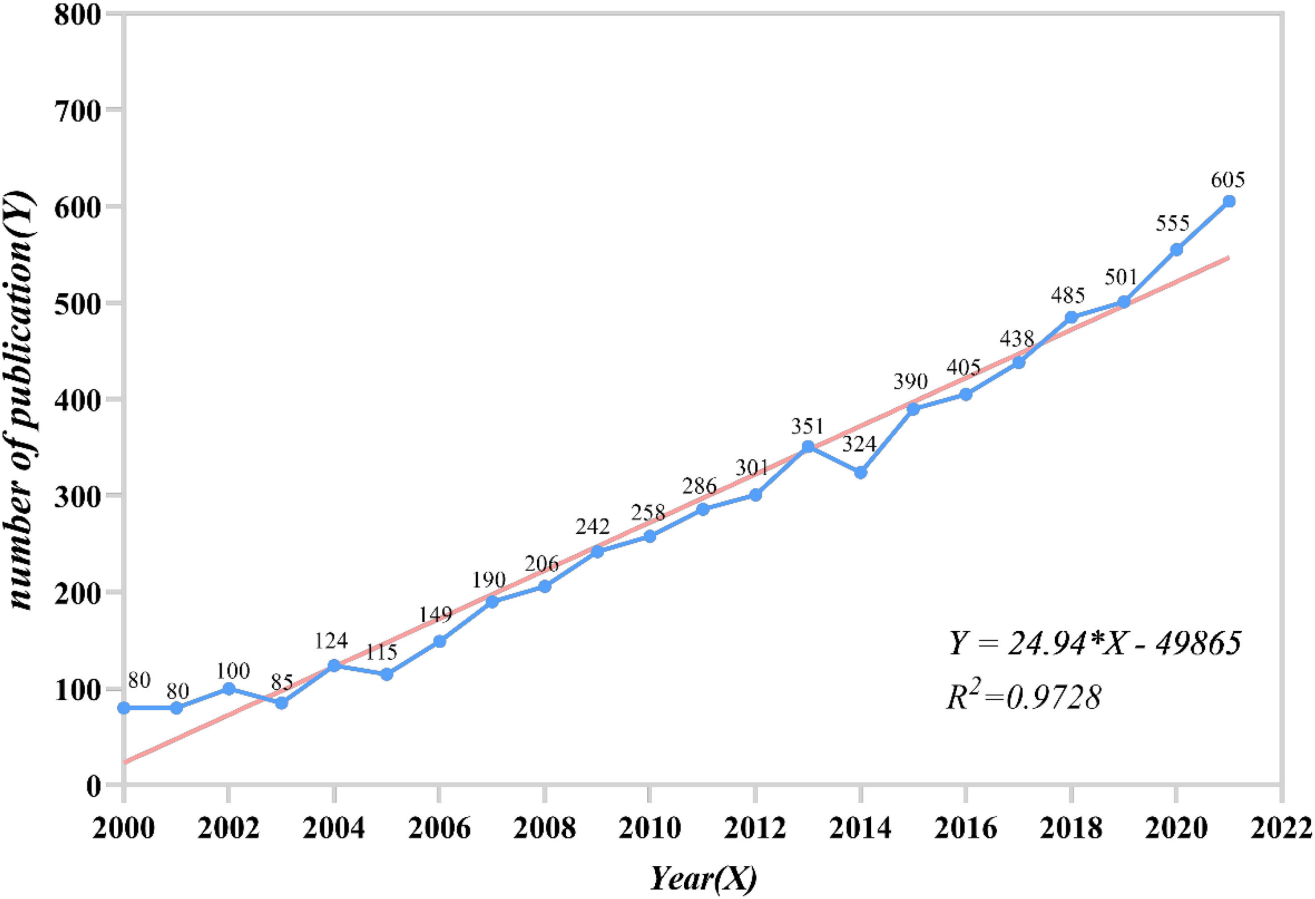
The number of published papers from 2000 to 2021.

The number of published papers on the research of life quality or psychological impact of gynecology oncology patients is shown in **Figure 2**. For the purpose of linear regression analysis, only publications from 2000 to 2021 are shown. In general, it has increased year by year. It can be seen that the rise slowed down in 2000–2003, while in recent years (2019–2021), the rise has been rapid, with an increase of about 50 papers per year.

### 3.2 Geographical distribution of the publications

**Figure 3:**
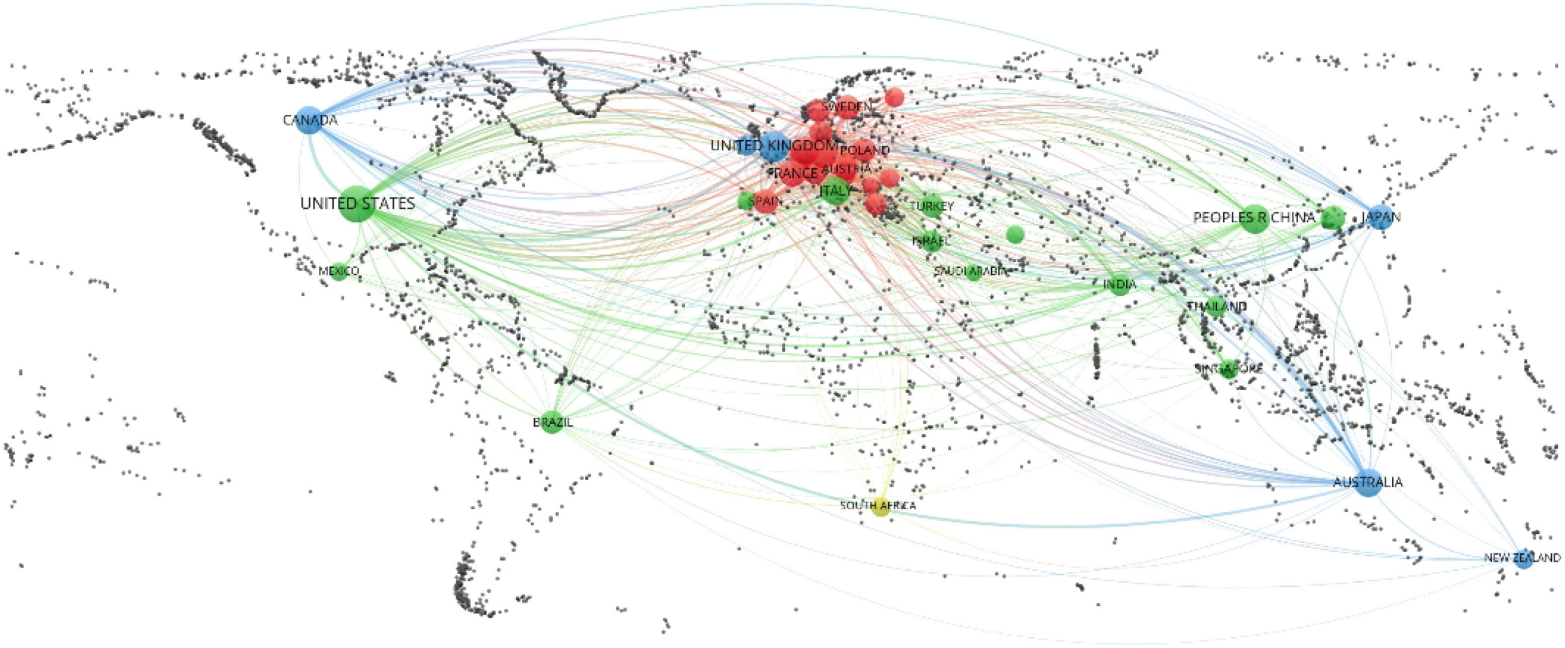
The geographic visualization analysis of country collaboration based on the co-authorship analysis of VOSviewer (Taiwan is annexed to the People’s Republic of China; England, Scotland, Northern Ireland, and Wales were incorporated into the United Kingdom).

The geographic visualization analysis of country collaboration based on the co-authorship analysis of VOSviewer and RStudio is shown in **Figure 3**. To visualize clearly, only a minimum number of documents, not more than 20, were included in this map, which contained 38 countries. The top 10 most relevant countries according to the corresponding author’s country are listed in **Table 1**. The listed countries published a total of 5,031 articles, accounting for 77.5% of the total. The United States ranked first (n = 2,075, 32.0%), followed by China (n = 572, 8.8%), the United Kingdom (n = 509, 7.9%), Australia (n = 342, 5.3%), Italy (n = 340, 5.2%), and so on. **Table 1** also shows single-country publications (SCPs), multiple-country publications (MCPs), and MCP ratio (MCP/articles). France is the country with the highest MCP ratio (0.344), and Japan is the country with the lowest (0.052) in the top 10 list.

**Table 1:** Top 10 most relevant countries by corresponding authors.

Rank	Country	Articles	SCP^a^	MCP^b^	Frequency	MCP ratio^c^
1	United States	2,075	1,829	246	0.32	0.119
2	China	572	502	70	0.088	0.122
3	United Kingdom	509	365	144	0.079	0.283
4	Australia	342	249	93	0.053	0.272
5	Italy	340	279	61	0.052	0.179
6	Canada	292	219	73	0.045	0.25
7	Netherlands	292	237	55	0.045	0.188
8	Germany	248	187	61	0.038	0.246
9	Japan	210	199	11	0.032	0.052
10	France	151	99	52	0.023	0.344

a Single-country publications, indicating that the authors of the publications are from the same country.

b Multiple-country publications, indicating that the authors of the publications are from multiple countries, which means the cooperation between countries.

c MCP/articles, indicating the ratio of co-authored articles in the country’s published articles.

### 3.3 The distribution of institutions

**Figure 4:**
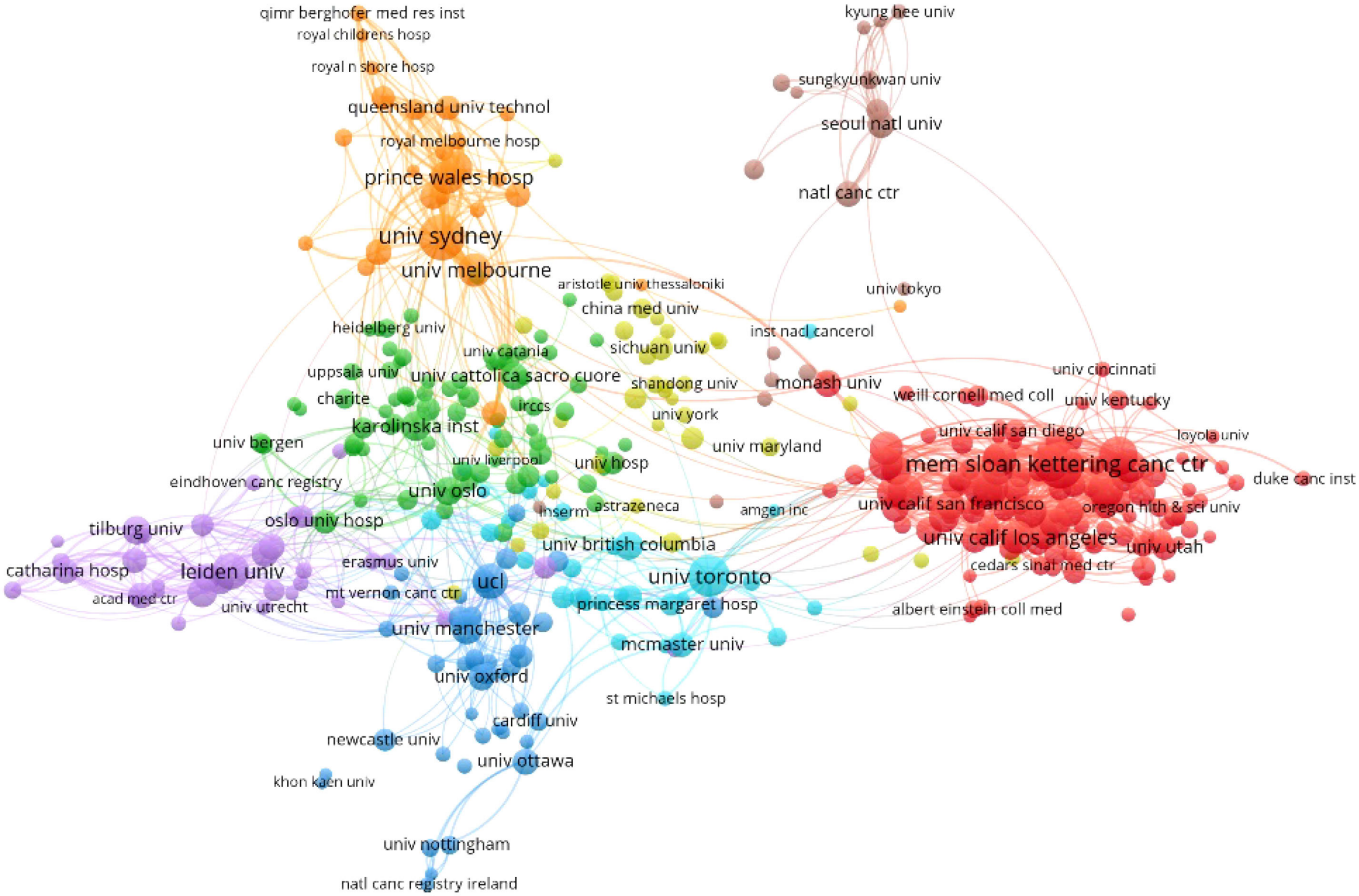
The co-authorship of the institution network.

The institution network based on the co-authorship analysis is listed in **Figure 4**.

In this network, only a minimum number of documents of not more than 10 were included, containing 390 organizations. The map shows the largest set of connected items consisting of 389 notes. It contains eight clusters.

The top 10 institutions that contributed to the publications are listed in **Table 2**. As we can see, most top 10 affiliations came from the United States (n = 6) and one each from Australia, the United Kingdom, Canada, and the Netherlands. The University of Sydney contributes the most (n = 167) in this field, followed by Memorial Sloan Kettering Cancer Center (n = 166) and The University of Texas MD Anderson Cancer Center (n = 164). Memorial Sloan Kettering Cancer Center contributed the most citations (n = 8,399).

**Table 2:** The top 10 institutions that contributed to the publications.

Rank	Affiliation	Country	Articles	Citations
1	The University of Sydney	Australia	167	6,111
2	Memorial Sloan Kettering Cancer Center	United States	166	8,399
3	The University of Texas MD Anderson Cancer Center	United States	164	5,511
4	University of Toronto	Canada	133	7,631
5	Ohio State University	United States	112	5,821
6	University Leiden	Netherlands	104	5,744
7	Northwestern University	United States	101	4,269
8	University College London	United Kingdom	96	4,068
9	Harvard University	United States	94	7,161
10	University of Pennsylvania	United States	92	3,862

### 3.4 Journals

**Figure 5:**
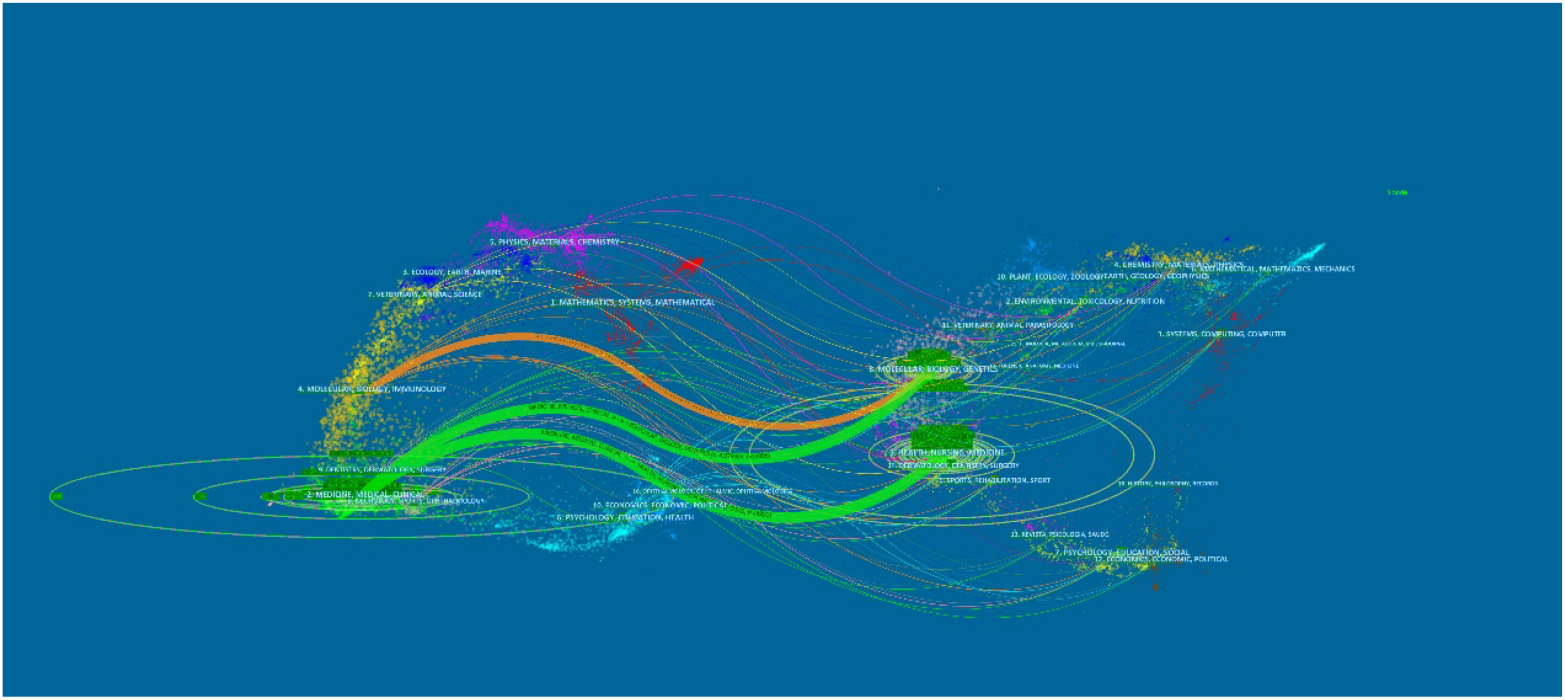
The dual-map overlay of journals that contributed to publications.

**Figure 5** shows the dual-map overlay of journals, with the citing journal in which source articles were published on the left and the cited journal in which references were published on the right. Each spline curve started from a citing journal and pointed to a cited journal. Citing and cited journals are marked with ovals (25). Two green paths and one orange path are demonstrated as main citation paths through z-score analysis. The green paths present that the publications from the medicine/medical/clinical fields were most citing the health/nursing/medicine fields of journals (z = 9.13, f = 24,980) and the molecular/biology/genetics fields of journals (z = 4.98, f = 13,896). The orange path presents that the publications from the molecular/biology/genetics fields were most cited by the molecular/biology/immunology fields of journals (z = 1.67, f = 5042).

**Table 3** lists the top 10 most relevant journals and most cited journals. *Gynecologic Oncology* contributed the highest publication numbers (n = 469). Among the top 10 relevant lists, the *Journal of Clinical Oncology* has the highest impact factor (IF = 50.769), and it also ranked first in the top 10 most cited journals (n = 18,100). The journal with the highest IF in the cited source list was the *Lancet* (IF = 202.731), followed by the *New England Journal of Medicine* (IF = 176.079) and *JAMA-Journal of the American Medical Association* (IF = 157.335). Moreover, it could be seen that four journals are in both the most relevant list and most cited list, namely, *Gynecologic Oncology*, *Cancer*, *International Journal of Gynecological Cancer*, and *Journal of Clinical Oncology*.

**Table 3:** Most relevant journals and most cited journals.

Rank	Sources	Articles	IF	JCR category (quartile)
Most relevant sources				
1	*Gynecologic Oncology*	469	5.304	Oncology – SCIE (Q2); obstetrics and gynecology – SCIE (Q1)
2	*International Journal of Gynecological Cancer*	256	4.678	Oncology – SCIE (Q2); obstetrics and gynecology – SCIE (Q1)
3	*Supportive Care in Cancer*	156	3.359	Oncology – SCIE (Q3); health care sciences and services – SCIE (Q2); rehabilitation – SCIE (Q1)
4	*Psycho-Oncology*	140	3.955	Psychology, multidisciplinary – SSCI (Q2); social sciences, biomedical – SSCI (Q2); oncology – SCIE (Q3); psychology – SCIE (Q2)
5	*Journal of Clinical Oncology*	104	50.769	Oncology – SCIE (Q1)
6	*Cochrane Database of Systematic Reviews*	93	12.008	Medicine, general and internal – SCIE (Q1)
7	*BMC Cancer*	78	4.638	Oncology – SCIE (Q2)
8	*Cancer*	77	6.921	Oncology – SCIE (Q1)
9	*Archives of Gynecology and Obstetrics*	60	2.493	Obstetrics and gynecology – SCIE (Q3)
10	*PLoS One*	60	3.752	Multidisciplinary sciences – SCIE (Q2)
Most cited sources				
1	*Journal of Clinical Oncology*	18,100	50.769	Oncology – SCIE (Q1)
2	*Gynecologic Oncology*	16,416	5.304	Oncology – SCIE (Q2); obstetrics and gynecology – SCIE (Q1)
3	*New England Journal of Medicine*	6,208	176.079	Medicine, general and internal – SCIE (Q1)
4	*International Journal of Gynecological Cancer*	4,849	4.678	Oncology – SCIE (Q2); obstetrics and gynecology – SCIE (Q1)
5	*Lancet*	4,499	202.731	Medicine, general and internal – SCIE (Q1)
6	*Cancer*	4,386	6.921	Oncology – SCIE (Q1)
7	*JAMA-Journal of the American Medical Association*	4,167	157.335	Medicine, general and internal – SCIE (Q1)
8	*British Journal of Cancer*	3,773	9.089	Oncology – SCIE (Q1)
9	*International Journal of Radiation Oncology Biology Physics*	3,699	8.013	Oncology – SCIE (Q1); radiology, nuclear medicine and medical imaging – SCIE (Q1)
10	*Obstetrics and Gynecology*	3,564	7.623	Obstetrics and gynecology – SCIE (Q1)

IF, impact factor, based on the 2020 Journal Citation Reports 2020 from Clarivate Analytics; JCR, Journal Citation Reports.

### 3.5 Top cited publications and reference burst

**Figure 6:**
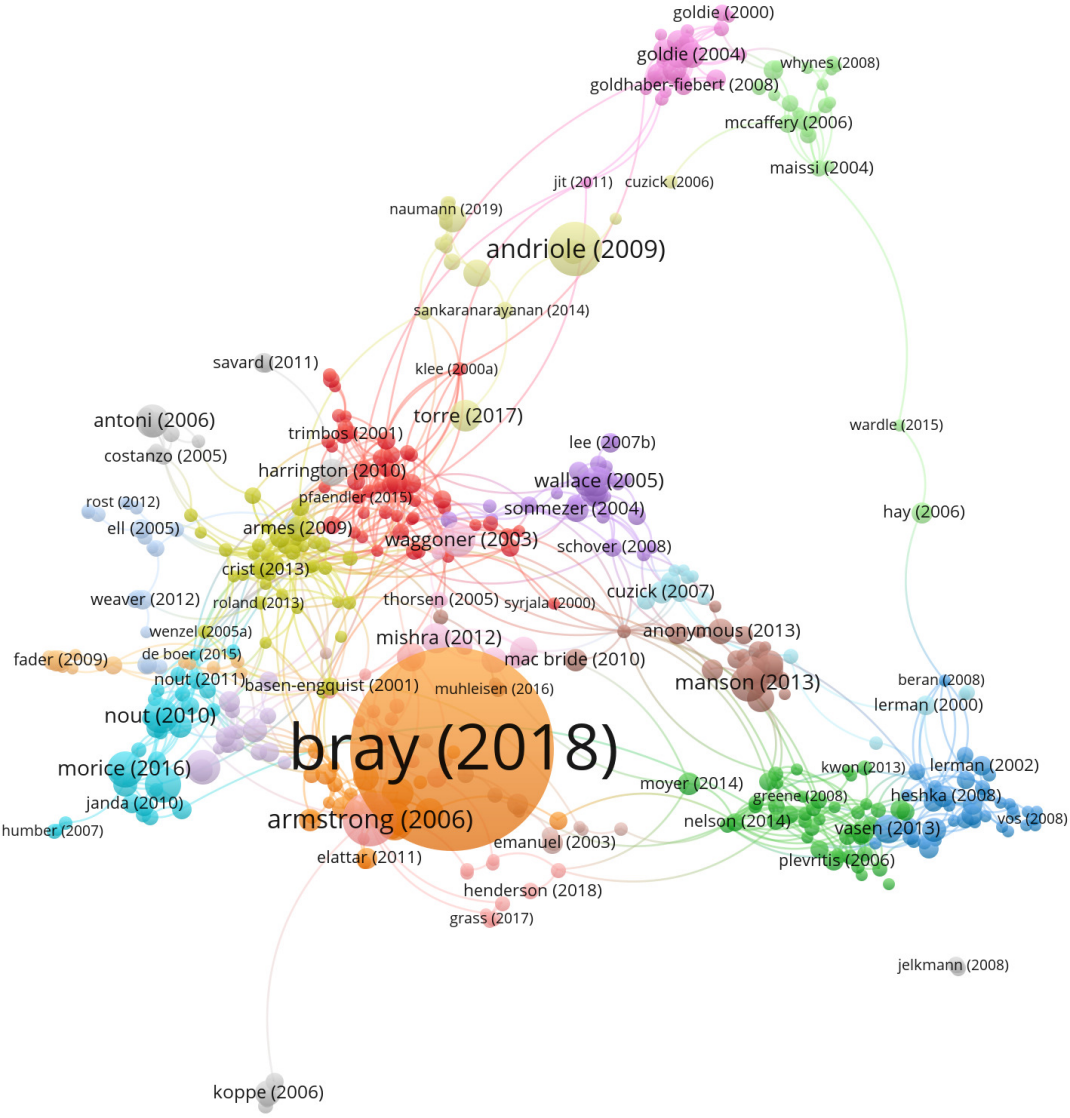
The citation of document network.

The citation of the document network is shown in **Figure 6**. The network was based on the number of times they cited each other. There are 748 citations in this document, which meets our minimum requirement of 60. Finally, the largest set of connected items consisted of 496 items and 22 clusters. **Table 4** lists the top 20 cited papers according to local citations. The global citation, which means the citation based on the Web of Science database, was also listed in **Table 4**. The article (Global Cancer Statistics 2018: GLOBOCAN estimates of incidence and mortality worldwide for 36 cancers in 185 countries) written by Bray F et al. (26) in 2018 had the highest citation both locally (n = 149) and globally (n = 42,425). The top 12 had a local citation of more than 100 times.

**Table 4:** Top 20 locally cited publications in QoL or mental health of gynecologic oncology patients.

Rank		Title	First author	Journal	Year	Local citations	Global citations		Reference	
1		Global cancer statistics 2018: GLOBOCAN estimates of incidence and mortality worldwide for 36 cancers in 185 countries	Bray F	*CA-A Cancer Journal for Clinicians*	2018	149	42,425		(26)
2		The European Organization for Research and Treatment of Cancer (EORTC) Quality-of-Life questionnaire cervical cancer module: EORTC QLQ-CX24	Greimel ER	*Cancer*	2006	129	166		(27)
3		Intraperitoneal cisplatin and paclitaxel in ovarian cancer	Armstrong DK	*New England Journal of Medicine*	2006	128	1,959		(28)
4		Reliability and validity of the functional assessment of cancer therapy-ovarian	Basen-Engquist K	*Journal of Clinical Oncology*	2001	122	180		(29)
5		Longitudinal study of sexual function and vaginal changes after radiotherapy for cervical cancer	Jensen PT	*International Journal of Radiation Oncology, Biology, Physics*	2003	110	219		(30)
6		An international field study of the reliability and validity of a disease-specific questionnaire module (the QLQ-OV28) in assessing the quality of life of patients with ovarian cancer	Greimel E	*European Journal of Cancer*	2003	109	149		(31)
7		Quality of life in long-term cervical cancer survivors	Wenzel L	*Gynecologic Oncology*	2005	109	157		(32)
8		A critical review of patient-rated quality of life studies of long-term survivors of cervical cancer	Vistad I	*Gynecologic Oncology*	2006	106	142		(33)
9		Quality of life and sexual problems in disease-free survivors of cervical cancer compared with the general population	Park SY	*Cancer*	2007	104	149		(34)
10		Long-term psychological impact of carrying a BRCA1/2 mutation and prophylactic surgery: a 5-year follow-up study	Van Oostrom I	*Journal of Clinical Oncology*	2003	103	202		(35)
11		Vaginal brachytherapy versus pelvic external beam radiotherapy for patients with endometrial cancer of high-intermediate risk (PORTEC-2): an open-label, non-inferiority, randomised trial	Nout RA	*Lancet*	2010	102	725		(36)
12		Early-stage cervical carcinoma, radical hysterectomy, and sexual function. A longitudinal study	Jensen PT	*Cancer*	2004	101	202		(37)
13		Quality-of-life effects of prophylactic salpingo-oophorectomy versus gynecologic screening among women at increased risk of hereditary ovarian cancer	Madalinska JB	*Journal of Clinical Oncology*	2005	98	173		(38)
14		Post-treatment sexual adjustment following cervical and endometrial cancer: a qualitative insight	Juraskova I	*Psycho-Oncology*	2003	88	168		(39)
15		Resilience, reflection, and residual stress in ovarian cancer survivorship: a gynecologic oncology group study	Wenzel LB	*Psycho-Oncology*	2002	86	182		(40)
16		Quality of life and sexual functioning after cervical cancer treatment: a long-term follow-up study	Greimel ER	*Psycho-Oncology*	2009	79	124		(41)
17		Health-related quality of life in cervical cancer survivors: a population-based survey	Korfage IJ	*International Journal of Radiation Oncology, Biology, Physics*	2009	78	102		(42)
18		Psychological impact of human papillomavirus testing in women with borderline or mildly dyskaryotic cervical smear test results: cross sectional questionnaire study	Maissi E	*BMJ-British Medical Journal*	2004	73	149		(43)
19		Psychological impact of genetic testing for cancer susceptibility: an update of the literature	Meiser B	*Psycho-Oncology*	2005	73	193		(44)
20		Quality of life and mental health in cervical and endometrial cancer survivors	Bradley S	*Gynecologic Oncology*	2006	72	107		(45)

QoL, quality of life.

**Figure 7:**
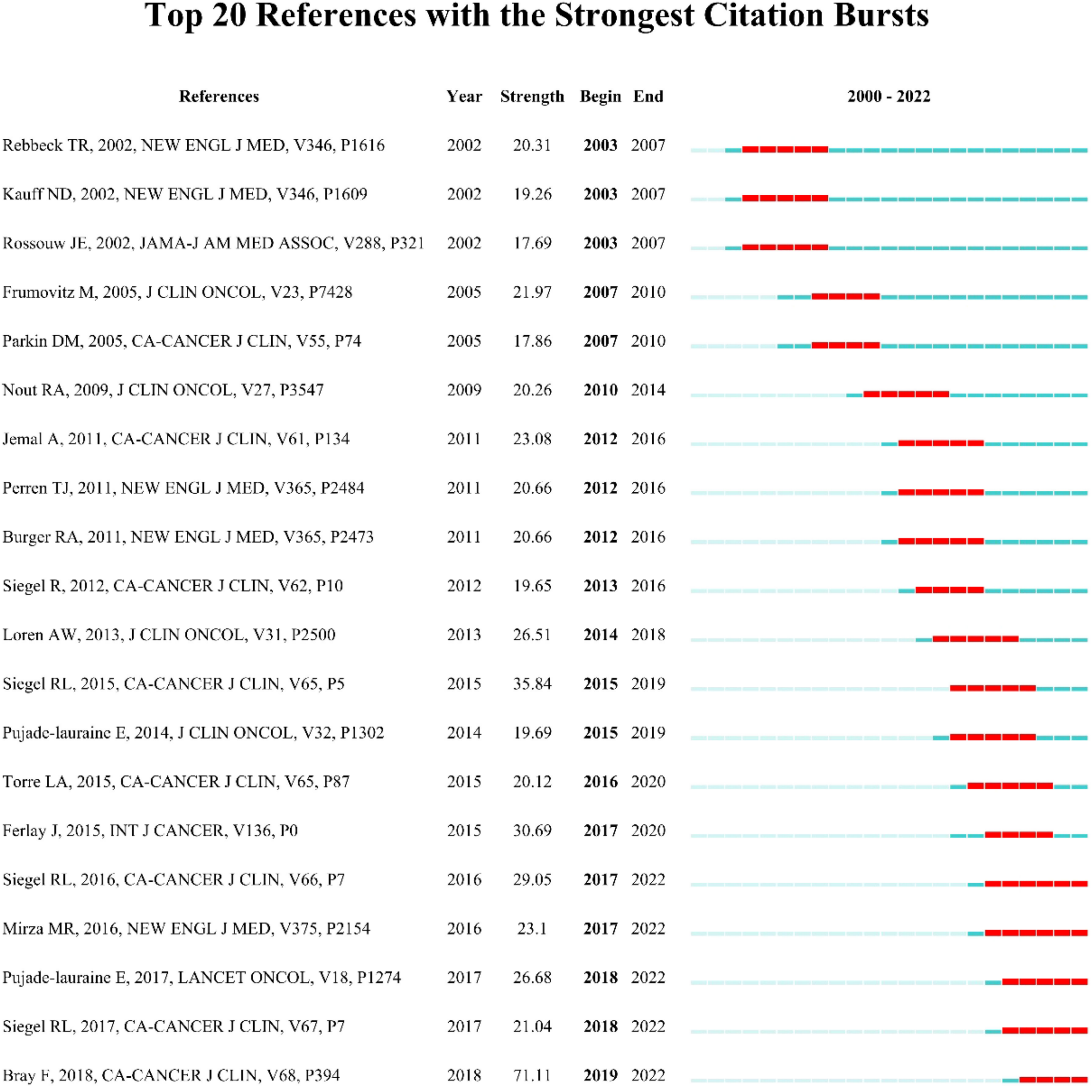
Top 20 references with the strongest citation bursts.

The top 20 references with the strongest citation bursts are shown in **Figure 7**. The basic meaning of burst detection is that the value of variable changes significantly in a short period of time. The reference with the strongest bursts was published by Bray F et al. in 2018 (strength = 71.11) followed by Siegel RL et al. in 2015 (strength = 35.84) and Ferlay J et al. in 2015 (strength = 30.69). Notably, the article written by Bray F et al. was also with the highest local and global citation numbers listed before. The citation bursts began in 2003 with Rebbeck TR et al. (from 2003 to 2007), Kauff ND et al. (from 2003 to 2007), and Rossouw JE et al. (from 2003 to 2007). The latest citation burst was detected in 2019 due to the article published by Bray F et al. (from 2019 to 2022).

### 3.6 Contributions of authors

**Figure 8:**
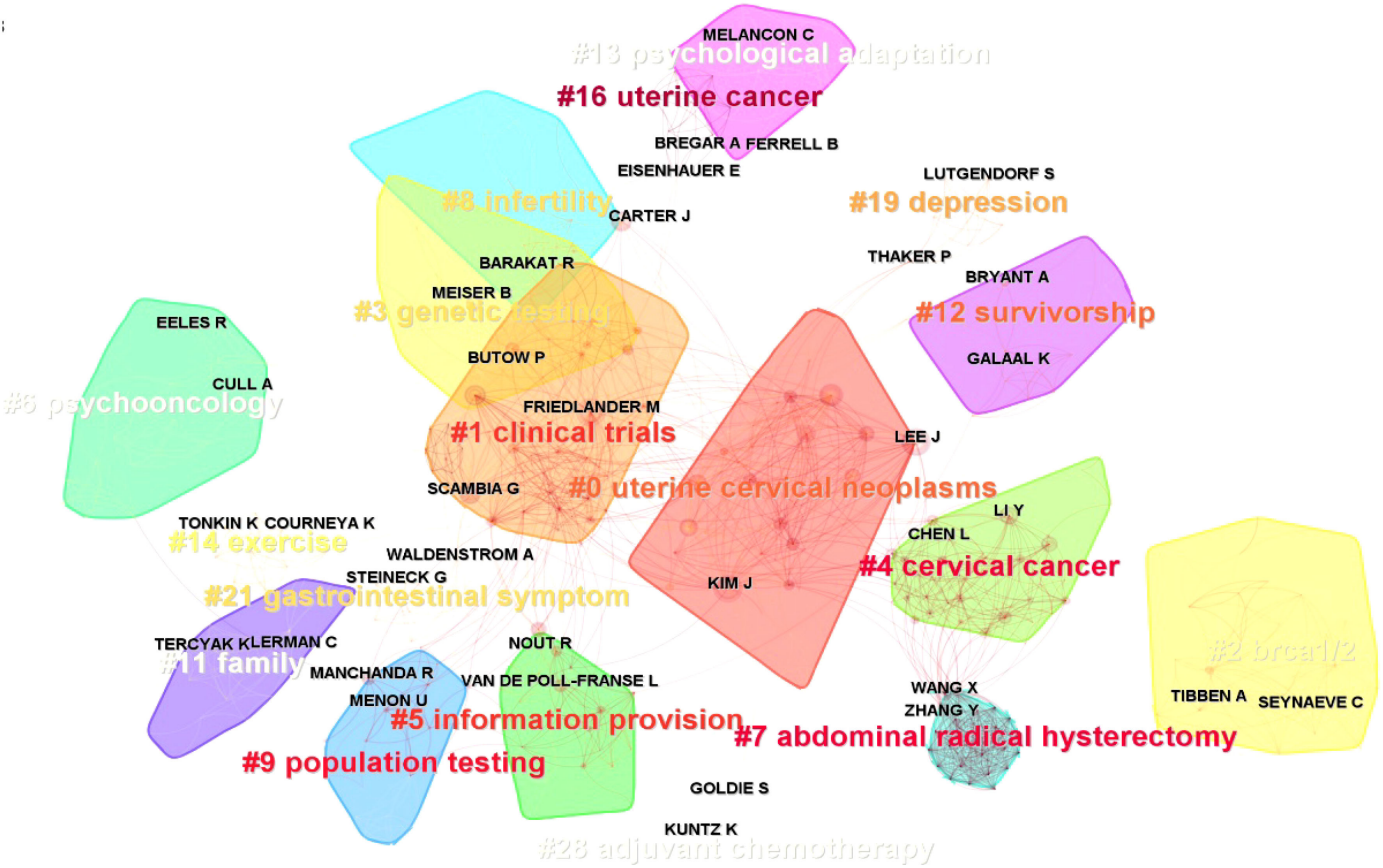
The network of authors involved in quality of life (QoL) or mental health of gynecologic oncology patients.

The network of authors and the cluster by keywords are presented in **Figure 8**. The modularity Q = 0.8285 (Q > 0.3 implies a reasonable clustering structure), and the weighted mean silhouette S = 0.9413 (S > 0.5 means reasonable clustering, and S > 0.7 implies convincing clustering). The largest cluster, #0, was tagged as uterine cervical neoplasms. It could be seen that authors Kim J and Lee J were in this cluster. **Table 5** lists the top 10 relevant and most co-cited authors. Kim J was the most productive author with 63 publications, followed by Friedlander M (61 publications) and Lee J (55 publications). The author co-citation analysis of cited paper showed that Aaronson NK ranks first with a count of 426, followed by Siegel RL (356) and Jemal A (334).

**Table 5:** Top 10 most productive authors and most co-cited authors.

Rank	Author	Year	Counts	Cited author	Year	Counts
1	Kim J	2008	63	Aaronson NK	2000	426
2	Friedlander M	2002	61	Siegel RL	2015	356
3	Lee J	2006	55	Jemal A	2002	334
4	Wenzel L	2002	51	Lerman C	2000	297
5	Scambia G	2008	50	Ferlay J	2010	272
6	Li Y	2008	44	Cella D	2001	269
7	Kim S	2012	44	Cella DF	2000	267
8	Cella D	2001	41	Greimel ER	2007	242
9	Sehouli J	2008	41	Ganz PA	2000	239
10	Carter J	2006	39	Rebbeck TR	2000	227

### 3.7 Keywords

**Figure 9:**
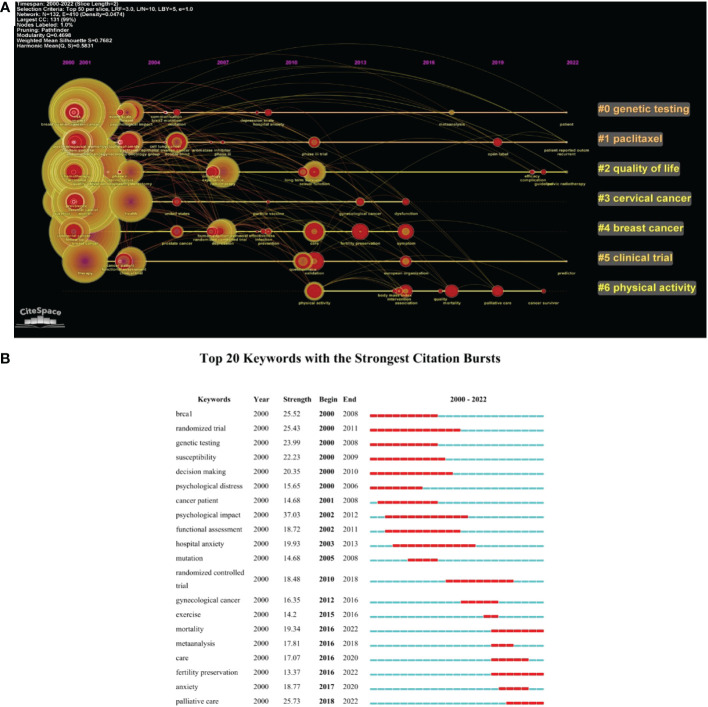
**(A)** The timeline view of the keywords (with citation bursts in red rings). **(B)** Top 20 keywords with the strongest citation bursts.

A cluster of keywords was made by CiteSpace V software and presented as a timeline view of keywords (**Figure 9A**). The timeline view could make the newly emerged keywords be recognized easily. The red circle in this figure shows the citation bursts. The clusters were labeled by #0 genetic testing, #1 paclitaxel, #2 quality of life, #3 cervical cancer, and so on. **Figure 9B** shows the top 20 keywords with the strongest citation bursts. The “psychological impact” had the strongest bursts (strength = 37.03, 2002–2012), followed by “palliative care” (strength = 25.73, 2018–2022). The keywords “mortality” (2016–2022), “fertility preservation” (2016–2022), and “palliative care” (2018–2022) have bursts till 2022.

## 4 Discussion

Our paper conducted a bibliometric analysis of QoL or psychological impact on gynecological cancer patients to show the status and hotspots in this field. The full record and cited references of 6,479 articles and reviews were included in our study from the WOSCC database. Bibliometrix R-package, VOSviewer, and CiteSpace V were applied to extract the items such as countries, institutions, journals, authors, references, and keywords; drew the collaboration network of these items; and pointed the citation bursts.

The United States was the most published country (n = 2,075), with about four times as many publications as China (n = 572, the second) and the United Kingdom (n = 509, the third). Among the top 10 countries, only China is a developing country. As for institutions, although the University of Sydney (Australia) ranked first with 167 papers, the following two institutions were from the United States with a similar number of publications as the top 1, Memorial Sloan Kettering Cancer Center—the world’s largest private cancer research center (n = 166)—and the University of Texas MD Anderson Cancer Center (n = 164). Moreover, six of the top 10 active institutions were from the United States. The other three institutions also came from developed countries, i.e., Canada, the Netherlands, and the United Kingdom. As we can observe, the distribution of countries and institutions was extremely unbalanced, and the United States dominated in this field. Institutions in those developed countries usually have sufficient funding and sound management. They can organize large clinical trial projects, and their patients may have high follow-up rates due to advanced online communication. In addition, most of the standardized scales for QoL or mental health were developed in western contexts (12), so it may be more difficult for developing countries to collect the exact perceptions of their patients.

The most cited publications listed in **Table 4** are basically all from 2000 to 2010, except for the first one (26), which was published in 2018. Articles published after 2011 may not have sufficient citations due to the short period of time rather than being less important. Citation bursts of reference could remedy this deficiency to some extent, as the bursts of references can show the active research topics with the time (46). The bursts of keywords can also reflect the trend in this field. The “randomized trial” had the widest year range of bursts (from 2000 to 2011). Then, it was gradually replaced by the related keyword “randomized controlled trial” (from 2010 to 2018). “Meta-analysis” had a burst from 2016 to 2018. The changes in these three keywords can be summarized as an evolution of the active article type in this field. The keywords “mortality”, “fertility preservation”, and “palliative care” are hotspot keywords will a duration until 2022. Mortality is mostly one of the most important endpoints in clinical trials, which deserves attention. The mortality rate of gynecological cancer patients is decreasing year by year substantially (1) (26). Several factors contribute to this, including primary prevention, widespread screening, increased accuracy of diagnosis, treatment efficacy, surgery, radiation and chemotherapy progress, and multidisciplinary treatment of patients (47).

Women of childbearing age who are diagnosed with gynecological cancer represent approximately 21% of those diagnosed (48). As survival rates of gynecologic cancer are increasing, the fertility preservation of reproductive-age women becomes an important part of improving QoL. Fertility-sparing surgical approaches and assisted reproductive technologies (ARTs) can be used as fertility preservation strategies (49). For patients who undergo radiation therapy as adjuvant therapy for fertility without having surgery, ovarian transposition and cryopreservation of oocytes or embryos are the preferred treatments (50). Many new techniques such as antiapoptotic/cell-preserving agents (51), and stem cell technologies (52) are being developed in fertility preservation. Palliative care focuses on providing specialized medical care to alleviate the symptoms and stress of patients suffering from serious illnesses (53). The palliative care team can work with the oncologists to provide support, manage symptoms, and plan advanced care. According to a comparative study, patients who died while in hospice care had an improved QoL when compared to those who were not in hospice care (54). Patients with gynecological cancer, symptom distress, and sexual functional status can also benefit from palliative care (55).

Nowadays, multiple effective treatments for gynecologic cancer have increased the survival rate of patients and improved their QoL. However, this cannot change the fact of incomplete reproductive organs, sexual dysfunction, the side effects of treatments, or the variable recurrence rate, which each patient of gynecological cancer has to face. The psychological impact caused by these facts can be heavy. Patients may suffer from stress, anxiety, depression, sexual dysfunction, and sleep deprivation (56). Many clinical trials have made efforts to improve the QoL and psychological health of their gynecologic cancer patients after treatment. However, two meta-analyses showed that psychosocial interventions (57) and exercise interventions (58) did not demonstrate improvements in QoL. Future studies are needed to explore possible strategies to improve QoL and psychological health after gynecologic cancer treatment.

Our study has some strengths: first of all, it is the first to perform a bibliometric analysis of QoL or psychological impacts of gynecologic cancer patients, and it provides a general view of this field and shows the hotspots and trends. In addition, this study used various bibliometric tools such as bibliometrix R-package, VOSviewer, and CiteSpace V, which enabled better visual analysis. However, there are also some limitations:

1. We only retrieved publications from the WOSCC database, and publications from other databases such as Scopus and PubMed were not included in our local dataset.
2. Only one author screened the publications and extracted information from the database, which may cause bias. However, these works were automatically generated through the website or map tools, which were not manually processed, so there was less chance of error.
3. Using the most cited publications to judge papers may have limits. The highest cited publication is Global Cancer Statistics (Bray F, 2018), which is less relevant to our topics, while some other valuable papers published recently may not be on our list due to the low citations.

## 5 Conclusion

Overall, our study provides a bibliometric analysis of QoL or psychological impact on gynecological cancer patients. For survivors of gynecologic cancer, attention should be focused on the recovery of their sexual function, the preservation of their fertility, and the maintenance of their feminine image as an important part of improving their QoL and promoting their psychological health. Our study can provide an overview and research hotspots in this field for future studies. It is hoped that further research will provide strategies for improving the QoL and psychological health of women with gynecologic cancer.

## Author contributions

JZ participated in data collection, data analysis, and drafting of the paper. YK participated in putting forward the idea, and the drafting and editing of the paper. YX and JY participated in the editing of the paper. All authors contributed to the article and approved the submitted version.
